# Direct and Indirect Targeting of HOXA9 Transcription Factor in Acute Myeloid Leukemia

**DOI:** 10.3390/cancers11060837

**Published:** 2019-06-17

**Authors:** Mélanie Lambert, Meryem Alioui, Samy Jambon, Sabine Depauw, Isabelle Van Seuningen, Marie-Hélène David-Cordonnier

**Affiliations:** 1 UMR-S1172 – JPArc – Centre de Recherche Jean-Pierre Aubert Neurosciences and Cancer, INSERM, F-59000 Lille, France; melanie-lambert89@live.fr (M.L.); meryem.alioui@inserm.fr (M.A.); samy.jambon@gmail.com (S.J.); sabine.depauw@inserm.fr (S.D.); isabelle.vanseuningen@inserm.fr (I.V.S.); 2 Université de Lille, F-59000 Lille, France; 3 CHU Lille, F-59000 Lille, France; 4 Institut pour la Recherche sur le Cancer de Lille, F-59045 Lille, France

**Keywords:** HOXA9, acute myeloid leukemia, transcription factor, epigenetic, protein/protein interaction inhibitors, protein/DNA interaction inhibitors

## Abstract

HOXA9 (Homeobox A9) is a homeotic transcription factor known for more than two decades to be associated with leukemia. The expression of HOXA9 homeoprotein is associated with anterior–posterior patterning during embryonic development, and its expression is then abolished in most adult cells, with the exception of hematopoietic progenitor cells. The oncogenic function of HOXA9 was first assessed in human acute myeloid leukemia (AML), particularly in the mixed-phenotype associated lineage leukemia (MPAL) subtype. HOXA9 expression in AML is associated with aggressiveness and a poor prognosis. Since then, HOXA9 has been involved in other hematopoietic malignancies and an increasing number of solid tumors. Despite this, HOXA9 was for a long time not targeted to treat cancer, mainly since, as a transcription factor, it belongs to a class of protein long considered to be an “undruggable” target; however, things have now evolved. The aim of the present review is to focus on the different aspects of HOXA9 targeting that could be achieved through multiple ways: (1) indirectly, through the inhibition of its expression, a strategy acting principally at the epigenetic level; or (2) directly, through the inhibition of its transcription factor function by acting at either the protein/protein interaction or the protein/DNA interaction interfaces.

## 1. Introduction

Transcription factors represent a large class of proteins with more than 1500 members, of which 15%–20% are considered oncogenes [1,2]. They are classified into more than 70 different families based on sequence and structure homologies of their DNA-binding domains. Among them, the homeobox DNA binding domain (homeodomain, HD) family encompasses more than 250 transcription factors, themselves subdivided into 21 different sub-families, including HOX-Like, Para-HOX, NK-Like, TALE (three amino acid loop extension) containing the PBC and MEIS groups among other), POU, HNF, LIM, Paired (PRD) and PRD-like subfamilies [3,4]. Derived from *Antennapedia* and *Bithorax* ancestors, the HOX-Like subgroup corresponds to the HOX cluster genes and is the only group of HD proteins conventionally named “HOX genes.” Organized into four paralog clusters in animals, the number and identity of HOX genes varies depending on the species. In humans, 39 HOX proteins are organized from 1 to 13 (as originally defined in *Drosophila*) in the four paralog clusters A to D. After their spatiotemporal expression, critical for patterning of the anterior–posterior axis in embryos, HOX gene expression is largely repressed in adults but controlled reactivation allows dynamic expression in adults to lead or reactivate some cellular processes, including hematopoiesis [5,6,7,8], wound healing [9,10], vascularization, endometrial development/fertility [11,12,13,14,15] and many other processes of body repair and homeostasis [16,17,18,19,20]. In particular, HOXA9 expression is finely controlled and decreases during the progression of normal hematopoiesis [21].

A large proportion of HOX cluster genes are considered oncogenes due to their implication in translocations, mutations or improper expression dynamics in some tissues. They are associated with cancer at the initiation, development or metastasis stages and are over-expressed in cancer cells relative to normal tissue (for reviews [22,23,24,25,26,27,28,29]). As an example, the oncogenic function of HOXA cluster genes was originally well described in hematopoietic disorders. This is particularly the case of HOXA9 in acute myeloid leukemia (AML).

In order to be highlighted as a target for clinical development against cancer, a transcription factor would have to evidence oncogene addiction in the pathology, which may be reflected by its over-expression in the pathology and by its inhibition, which is supposed to abolish one or more of the cancer cell properties, such as proliferation, death inhibition and differentiation blockade. All of these points were evidenced for HOXA9 in AML as exemplified below.

## 2. HOXA9: A Leukemic Driver in AML

The leukemogenic function of HOXA9 was first assessed in the murine model BXH-2, a mouse strain that spontaneously develops AML through endogenous retroviral integration. Indeed, Hoxa9, as well as a number of other Hox genes and its co-factor Meis1 (myeloid ecotropic viral integration site 1), are frequently over-expressed in BXH-2 murine leukemic cells [30]. It was then evidenced that transplantation of cells over-expressing murine Hoxa9 by retroviral transduction evidenced a late onset of AML, a process that was accelerated by co-transduction with Meis1 [31]. 

In human leukemia, the first implication of HOXA9 was highlighted by the discovery of the NUP98-HOXA9 fusion protein resulting from t(7;11)(p15;p15) translocation [30,32], a rare (1%–3%) AML subtype associated with poor prognosis [33,34]. NUP98, a nucleoporin of 98 kDa, is a chaperone protein associated with the nuclear pore. NUP98-HOXA9 binds directly to DNA on the HOXA9-cognate sequence via HOXA9 homeodomain [35]. NUP98-HOXA9 chimera seems to induce myelodysplastic syndromes for a relatively long period before transformation in AML, a period that is reduced when MEIS1 is concomitantly expressed [36]. The NUP98-HOXA9 protein would also have a higher transcriptional activity than HOXA9 itself due to its highest stability (half-life three times longer) in relation to its resistance to ubiquitinylation mediated by CUL-4A that may partly explain its oncogenic function [37].

Besides expressed as an oncogenic fused protein, the implication of structurally unmodified HOXA9 as a leukemic driver was then evidenced as part of its over-expression in leukemic cells. Indeed, the relevance of HOXA9 expression in global survival of human leukemia patients was first demonstrated on gene expression signatures relating to patient outcome: HOXA9 was evidenced as the protein presenting the highest correlation with poor prognosis in a series of nearly 7000 genes [38]. This association of the level of HOXA9 expression with prognosis was also evaluated on an independent series of patients showing that low levels of HOXA9 (but also other HOXA and HOXB) gene expression is characteristic of a favorable cytogenetic AML subgroup [39,40]. Such general analyses now take into account the better knowledge of cytogenetic and molecular characteristics of different AML sub-types and it is now well established that HOXA9 over-expression is directly associated with some of them, themselves directly identified as good, intermediate, or adverse prognosis subgroups, with a total prevalence of ~70% of AML [41,42]. The main genetic alterations associated with HOXA9 over-expression in AML are presented in Table 1. 

The most described HOXA9-associated leukemias are: (1) acute leukemia (either myeloid or lymphoid) bearing MLL (mixed lineage leukemia, also called KMT2A) fusions [43,44,45,46,47], known as mixed phenotype acute leukemia (MPAL), and which represent ~5% of AML and are associated with poor prognosis; and (2) AML with nucleophosmin 1 (NPM1) mutations, which represent ~55% of normal karyotype AML and ~35% of all AMLs, and are associated with poor to intermediate prognosis depending on the nature of additional alterations, such as mutations of FLT3 kinase (Fms-like tyrosine kinase 3) [48,49,50].

**Figure 1:**
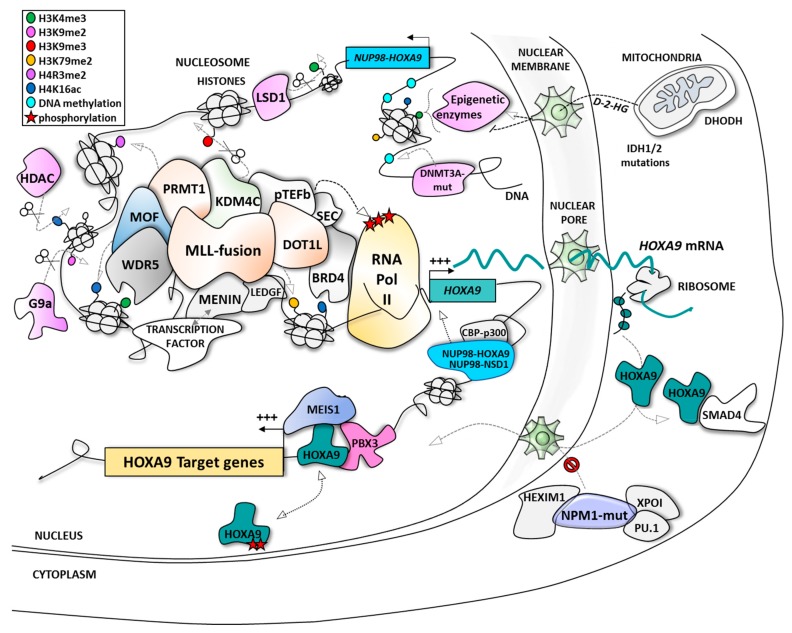
The different modes of regulation of HOXA9 expression and function in acute myeloid leukemia (AML). BRD4, bromodomain-related protein 4; CBP, CREB-binding protein; CDK9, cyclin-dependent kinase 9; D-2-HG, D-2-hydroxyglutarate; DHODH, dihydroorotate dehydrogenase; DNMT3A, DNA methyl transferase 3A; DOT1L, disruptor of telomeric silencing 1-like protein; HDAC, histone deacetylase; HEXIM1, hexamethylene bisacetamide (HMBA) inducible protein 1; HOXA9, homeobox A9; IDH, isocitrate dehydrogenase; KDM4C/lysine-specific demethylase 4C; LEDGF, lens epithelium-derived growth factor; LSD1, lysine-specific demethylase 1; MEIS1, myeloid ecotropic viral integration site 1; MLL, mixed lineage leukemia; MOF, males absent on the first; NPM1, nucleophosmin 1; NSD1, nuclear receptor binding SET domain protein 1; NUP98, nucleoporin 98kDa; PBX3, pre-B-cell leukemia transcription factor 3; PRMT1, protein arginine N-methyltransferase 1; pTEFb, positive transcription elongation factor b; SMAD4, mothers against decapentaplegic homolog 4; WDR5, WD repeat protein 5; XPO-1, exportin-1.

The AML subtype MPAL preferentially affects infants or is developed as a therapy-induced leukemia. MPAL is associated with poor prognosis with a five-year survival rate of less than 40% in infants compared to ~90% for non-MPAL [51]. The genomic breakpoints involve more than 130 different MLL translocation partners already described, with the 10 main partners representing >90% of the MLL translocations, including AF9 (~30%), AF10 (~16%), ELL (~10%), AF6 (~8%), and ENL (~6%) [52,53,54]. The major breakpoint cluster region is localized between exon 9 and intron 11 of the MLL gene in more than 80% of MPAL patients. These rearrangements generate a fusion between the N-terminal portion of the MLL protein containing its DNA binding domain and the carboxy-terminal portion of its protein partner [55]. The MLL protein will lose its SET domain and its domain for binding to ASB2, a ubiquitin ligase causing its proteolysis. Thereby, the fusion proteins generated will no more be degraded [56]. Interestingly, the main translocation partners (AF9/AF10/ENL), as well as minor partners such as AF4, are proteins that normally function within a large protein complex associated with the MLL protein (within a large complex or different sub-complexes). Translocations seem to physically fix proteins together in order to favor the stability and functionality of the MLL complex, particularly through interaction (direct or indirect) with the disruptor of telomeric silencing 1-like protein DOT1L (through direct interaction with AF10, for instance), an epigenetic partner that methylates lysine-79 residues of histone H3 proteins as a transcriptional activation mark [57,58,59], or with p-TEFb kinase (through direct interaction with AF4, for instance) that phosphorylates RNA polymerase II to allow gene transcription [60]. Among other proteins implicated in the active MLL complex are Menin [61,62], LEDGF (lens epithelium-derived growth factor) [61,63], WDR5 (WD repeat protein 5) [64], BRD4 (bromodomain-related protein 4) [65], HDAC (histone deacetylase) [66,67], KDM4C/JMJD2C (lysine-specific demethylase 4C/jumonji domain-containing protein 2C) and PRMT1 (protein arginine N-methyltransferase 1) [68] (Figure 1).

In mice, grafting of bone marrow cells with retroviral transduction of MLL fusion proteins deregulated the expression of Hox genes [69]. All MPAL patients not only evidenced HOXA9 over-expression but also middle HOXA cluster over-expression, as exemplified by MLL-AF9 fusion, which positively regulates the expression of HOXA6, HOXA7, HOXA9, and HOXA10 [70]. MLL translocations also increase the expression of MEIS1, which is generally positively correlated with HOXA9 expression [29]. In addition, although the role of an alternative transcript HOXA9T is not clearly defined, HOXA9T is over-expressed in AMLs with MLL arrangements [71]. Beside these fusion proteins, partial tandem duplications (MLL-PTD) were discovered, particularly in de novo AML cases. The most common duplication event is a copy of exons 5-11 or 5-12 inserted into intron 4 and resulting in the replication of the N-terminal portion of MLL that contains the AT-hook DNA binding domain. These alterations represent 12% of AML [53] and are associated with poor prognosis. By contrast, MPAL with MLL-PTD evidences moderate up-regulation of HOXA9 expression [72]; however, HOXA9 is still crucial for MLL-PTD-driven leukemogenic processes [42,73]. 

**Figure 2:**
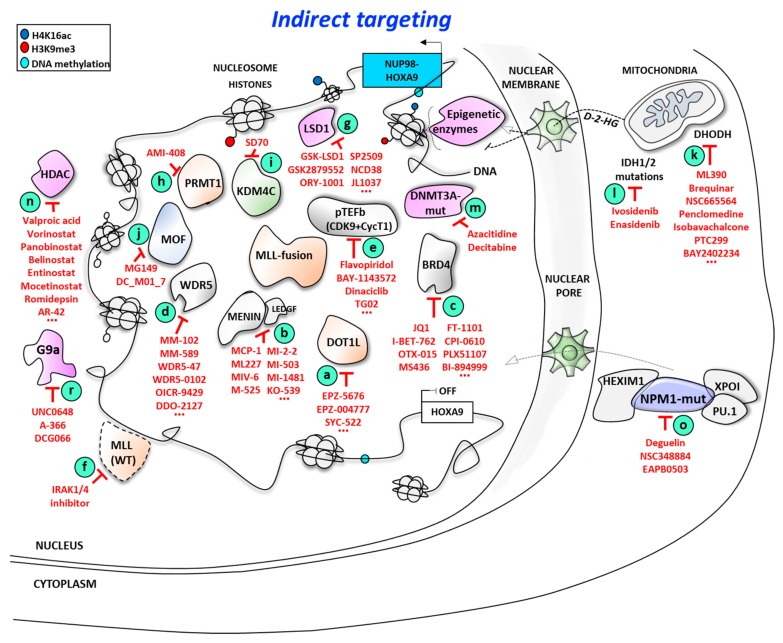
Indirect targeting of HOXA9 expression in AML: multiple epigenetic and non-epigenetic therapeutic opportunities.

The NPM1 mutations are generally present in de novo adult AML with normal karyotype and were evidenced to be correlated with the over-expression of HOXA, MEIS1, and FLT3 genes [48,74]. Under normal conditions, NPM1 chaperone protein is located in the nucleus. NPM1 mutations result in the delocalization of the protein into the cytoplasm causing over-expression of HOXA9, HOXA10, and MEIS1 [49] by a mechanism probably associating MLL, P-TEFb, DOT1L, and/or menin [74,75]. One of the suggested mechanisms for mutated-NPM1 (also called NPM1c+) control of HOXA9 expression is the activation of the transcriptional complex P-TEFb (positive transcription elongation factor b), a partner of the MLL complex usually sequestrated by HEXIM1 in the cytoplasm; HEXIM1 (hexamethylene bisacetamide (HMBA) inducible protein 1) sequestrated in the cytoplasm by mutated-NPM1 could no longer interact with P-TEFb, which could therefore activate the MLL complex and subsequently HOXA9 expression [76,77] (Figure 2). Recently, it was also highlighted that NPM1c+ leukemic cell survival requires upregulation of HOXA9 and its DNA-binding partner, the Pre-B-cell leukemia homeobox 3 PBX3 in a MLL/DOT1L dependent manner [75].

In parallel, Brunetti et al. demonstrated that HOXA (and HOXB) genes are not only direct downstream targets of NPM1c+ protein but also that the interaction between exportin-1 (XPO-1), a nuclear pore exporter, and NPM1c+ protein, maintains mutated-NPM1 in the cytoplasmic compartment as an important point explaining AML occurrence [78]. Moreover, Gu et al. [79] evidenced NPM1c+ interaction with PU.1/SPI1 transcription factor as another way to maintain NPM1 within this complex in the cytoplasm, whereas PU.1 over-expression is associated with a decrease in HOXA gene expression. 

The over-expression of HOXA9 is also found in many NUP98 fusions containing AML samples, accounting for a total of 1%–3% of AML [33]. Some examples are presented in Table 1. A lot of these partners are associated with epigenetic control or the DNA binding function (with, for instance, a lot of homeodomain containing transcription factor partners). The most frequent of these is the H3K36 methyltransferase NSD1 (nuclear receptor binding SET domain protein 1), forming the NUP98-NSD1 fusion protein that activates HOXA gene expression for leukemogenesis in 1%–2% AML [80].

Trisomy 8 (+8) represents ~10% of all AML and also correlates with a high level of HOXA9 expression [81]. Alone, trisomy 8 is not sufficient for leukemogenesis but is often associated with the t(7;12) or t(9;11) and t(1;11) MLL translocations. HOXA9 and HOXA10 are the first and second rated over-expressed genes in +8 AML, respectively, relative to normal bone marrow cells [81]. However, this analysis does not exclude MPAL and +8 double positive AML, and further analysis may be required to ensure that MLL alteration was not the main driver of HOXA9/10 over-expression in +8 AML.

In many other well-defined cytogenetic or molecular alterations associated with AML, HOXA9 can be frequently over-expressed but not in all patients of the same sub-group, suggesting that further analysis of additional alteration would be required. For instance, EVI1 over-expressing leukemia results from t(3;21)(q26;q22) associated with poor survival in 8%–10% AML and presents over-expression of the HOXA9 gene with a large spread of HOXA9 expression from positive to negative. 

In total, the proportion of HOXA9-over-expressed AML is ~70%. This high proportion highlights HOXA9 as an interesting potential target to treat such AML. 

The oncogenic function of HOXA9 in AML is associated with cell proliferation, differentiation blockade, increased malignancy of leukemic cells, and progenitor self-renewal maintenance [98]. Invalidation of HOXA9 expression in those cells impairs proliferation and leukemic properties, and re-activates differentiation processes, showing that HOXA9 is a functional target to restore differentiation in AML [47,99,100,101]. More precisely, the presence of the HOXA9 DNA binding domain is a prerequisite for HOXA9-induced leukemic transformation in mice models: (i) swapping HOXA9 homeodomain with HOXA1 homeodomain in the HOXA9 transcription factor is sufficient to abolish the leukemic potential of transduced murine hematopoietic progenitor cells engrafted in mice, whereas transferring HOXA9 homeodomain in the HOXA1 protein maintains the leukemic propensity of HOXA9 and results in common deregulated gene signatures with wild-type HOXA9-induced transformation [102]; (ii) mutating HOXA9 homeodomain at Asn51 to a serine residue (N51S) abolishes leukemic transformation in mice [103,104]. Similarly, HOXA9T, a splice variant protein which has lost its DNA binding domain, does not induce leukemia by itself, even if it seems to support the leukemogenic activity of HOXA9 [105] by a yet unclear mechanism of action, but may imply HOXA9T binding to transcription promoting factors such as CBP (CREB-binding protein) or some chaperone proteins [106]. Interestingly, the phosphorylation status of HOXA9 changes its DNA binding activity and consequently its propensity to induce leukemia, as demonstrated with protein kinase C (PKC) phosphorylation of Ser204 of the HOXA9 DNA binding domain, impairing DNA binding and leading to myeloid differentiation of murine Hoxa9-immortalized bone marrow cells [107].

In order to understand HOXA9-mediated leukemogenesis, the key point would be to identify the network underlying its transcriptional activity using ChIP-sequencing. However, due to the lack of ChIP and ChIP-seq grade antibodies directed against HOXA9 and other HOX proteins to identify endogenous targets for each HOX protein on the chromatin, most global ChIP analyses have used exogenous expression of tagged proteins. This is notably the case of a HA-tagged Hoxa9 protein in a Hoxa9- and Meis1-transformed murine bone marrow cell model to identify thousands of genomic binding regions of the murine Hoxa9 transcription factor, being associated or not with Meis. These studies identified several pro-leukemic Hoxa9 target genes such as Erg (ETS-related gene), Flt3, Lmo2 (LIM domain only 2), and c-Myb (myeloblastosis) [108]. However, a recent study has shown that Hoxa9 is a specific substrate of a granule protease and that its inhibition would allow the ChIP-sequencing analysis in primary transformed murine cells, showing a feedback loop driving expression of key oncogenes and cell cycle control genes [109].

Alternatively, microarray or RNA-seq analyzes have been successfully performed. Gene expression analyses in models over-expressing or interfering (shRNA, CRISPR9-Cas9) with HOXA9 expression showed significant transcriptomic modulations in which HOXA9 could act as an activator or a repressor, depending on target gene and cell context [110]. Most HOXA9-specific targets were also discovered individually, including Lmo2, Bcl-2, Fgf2, Igf1, Ink4a/b, and c-Myb [108,110,111,112,113,114,115,116]. In particular, HOXA9 functions as a pioneer factor at de novo enhancers and recruits CEBPα and the MLL3/MLL4 complex [117]. HOXA9 over-expression in progenitor cells, therefore, leads to significant enhancer reorganizations with prominent emergence of leukemia-specific de novo enhancers.

If HOXA9 could act alone to trigger leukemia, it requires cofactors to increase its propensity to induce leukemia. For HOXA9, the leukemogenic activity of MEIS1 was discovered through in vivo experiments in BXH-2 mice, a pro-viral insertion model in which 15% of induced AMLs are caused by pro-viral insertion into the Meis1 gene locus [118]. Like HOXA9, Meis1 expression decreases during normal differentiation of blood cells. In vivo, the presence of Hoxa9 expressed alone in murine bone marrow cells is not sufficient to rapidly induce leukemia (>6 months) whereas concomitant expression of Hoxa9 with Meis1 greatly shortened this period (approximately 67 days). However, Meis1 alone does not induce leukemia [31], Meis1 as an accelerator of Hoxa9-induced leukemia but not stricto sensu as an oncogene. In human AML samples, a correlation expression of MEIS1 and HOXA9 is observed, suggesting a parallel or common temporal action of these factors [30,119,120]. If MEIS1 potentiates the leukemia action of HOXA9, this is also the case of PBX3, whose expression is also strongly correlated with HOXA9 expression, especially in leukemia subtypes with a normal karyotype or associated with MLL rearrangements. The overexpression of PBX3 and HOXA9 thus favors the initiation and implantation of AML [29]. Inactivation of PBX3 and HOXA9 by down-regulating H3K79 methylation also represses NPM1c+ leukemic cell survival [75].

As HOXA9 is associated with a large proportion of AML, its inhibition is an interesting strategy against AML that could be achieved by different ways:

- inhibition of its expression;
- blockade of the specific protein/protein interaction crucial for its mechanism of action;
- or, more specifically as part of a transcription factor, the blockade of the interaction with its cognate sequence on the DNA.

## 3. Indirect Targeting of HOXA9 at the Expression Level

As presented above, it is well established that HOXA9, together with posterior HOXA genes, is regulated in MPAL at the expression level by epigenetic control driven by the oncogenic fused MLL proteins within a large epigenetic complex associating (simultaneously, sequentially, or as subgroups) the following proteins (Figure 2 and Table 2):

- DOT1L, which interacts with a large number of fused partners of MLL, themselves present in the non-oncogenic MLL complex;
- menin/LEDGF complex, which interacts with the N-terminal domain of MLL;
- WDR5, which interacts with the C-terminal part of MLL and binds to me-H3K4 marks during the transcription elongation process. Binding of WDR5 in complex with RbBP5 and ASH2L proteins with a WDR5 interacting motif (WIN) changes the conformation of the SET-domain of MLL to activate epigenetic function of the wild-type MLL complex;
- pTEFb complex, which makes contact with both MLL and DOT1L proteins and traps BRD4 to a large MLL-fusion complex.

However, it will lose interaction to other factors that bind to the C-terminal part of the wild-type MLL protein that is removed in MLL chimeras:

- MOF, which binds to the SET domain in the C-terminal part of MLL protein and acetylates H4K16 position at transcriptional initiation stage [121];
- UBE2O, which promotes wild-type MLL degradation in response to IL1 treatment and subsequent IRAK4 (interleukin-1 receptor-associated kinase 4) activation pathway [122] in order to remove the WT-MLL complex from the chromatin and thus to favor oncogenic MLL chimera complex binding to the chromatin.

In a more global view of the proteins interacting with MLL chimera complexes, also involved are the epigenetic regulators LSD1 (KDM1A) [123], HDAC [66,67], KDM4C (JMJD2C), or PRMT1 [68], which may also represent potential targets to abolish the MLL-driven expression of HOXA9 (Figure 2).

All these proteins and/or their protein/protein interaction interfaces represent interesting opportunities to inhibit the oncogenic processes of MLL fusion proteins on HOXA9 expression.

### 3.1. Targeting MLL-Interacting Partners at the Protein/Protein Interaction Level

Since MLL chimeras interact with multiple protein partners, different strategies were developed with the aim of decreasing HOXA +/- MEIS1 gene expression, restoring differentiation and, hopefully, consequently treating AML.

#### 3.1.1. Targeting Disruptor of Telomeric Silencing 1-like (DOT1L)/Mixed Lineage Leukemia (MLL) Proteins Interaction

The DOT1L histone methyltransferase (HMT) methylates lysine 79 of histone H3 (H3K79) and is aberrantly recruited at the MLL complex through interaction with the MLL fusion partner moieties, such as AF9 [124], AF4 [58], AF6 [65], AF10 [125], or ENL [126]. Inhibitors of DOT1L have been developed as competitors for DOT1L binding to MLL fusion proteins. The most advanced for therapeutic purpose is EPZ-5676 (pinometostat), which competes for S-adenosyl-methionine (SAM) interaction to the specific SAM-binding pocket of DOT1L, resulting in a conformational change that impairs HMT function. The benzimidazole-urea-containing nucleoside-like inhibitor EPZ-5676 is more effective (Ki ~0.08 nM) than the parental compound EPZ004777 (Ki ~0.3 nM) on DOT1L inhibition [127,128] and was chosen as the first-in-class HMT inhibitor to treat MPAL patients. Both EPZ-5676 and EPZ004777 cause a concentration-dependent decrease in HOXA9 and MEIS1 mRNA level expression, and induce cell death and differentiation of leukemic cells [129,130,131,132], but have also recently evidenced osteoclast differentiation as another way to favor the anti-leukemic process [133]. EPZ-5676 treatment correlates with an accumulation of cells in G0/G1 associated with a reduced proportion of cells in the S-phase and evidenced anti-leukemic activity in subcutaneous xenografts of MV4-11 cells expressing MLL-AF4 fusion from t(4;11) translocation [130]. Pinometostat was evaluated in phase I clinical trials against a series of 42 patients with relapsed/refractory MPAL (NCT01684150). Pinometostat treatment does not induce major toxicities and could be used safely at high doses. However, only a minor therapeutic response was reported when used as a single agent with only two MPAL patients showing complete remission, both resulting from translocation t(11;19), leading to MLL-ELL chimera for one and MLL-ENL fusion for the other [134]. Based on its poor toxicity and first positive results, it is proposed that additional clinical trials would be required to evaluate pinometostat not as a single therapy but in combination with a validated drug such as Ara-C or azacytidine, which are two drugs that evidenced synergic anti-leukemic activities in co-treatment with EPZ-5676 in cellular models [135].

Interestingly, DOT1L inhibitor treatments of AML with MLL-PTD [136], mutated-DNMT3A [132] and mutated-NPM1 [74,75] also present interesting results. The DOT1L inhibitor efficiency on mutated-NPM1 might partially be explained by an increase in the level of DOT1L protein (but not mRNA) in mutated-NPM AML [75] and by the presence of both DOT1L and NPM1 proteins within a proteic complex as identified by tandem affinity purification and mass spectrometry analysis [137]. 

Other DOT1L inhibitors (Figure 2, point **a**; Table 2) were also developed, including SYC-522 as a highly selective DOT1L inhibitor with Ki ~0.5 nM. SYC-522 was efficient in vivo in a MLL-AF4 fusion cell model (MV4-11) but not in a MLL-AF9 fusion model (MOLM-13) even if, in both cell models, SYC-522 reduced the levels of HOXA9 and MEIS1 expression by ~50% and induced differentiation as evidenced by an accumulation of the G0/G1 cell population and by an increase in CD14-positive cells [138].

This is, for instance, also the case for:

- the pyrimidyl-aminoquinoline derivative **9e**, that interacts with DOT1L at the µM range and reduces HOXA9 and MEIS1 expression [139];
- compounds **3** and **9,** identified by high throughput screening [140];
- DC_L115, which binds DOT1L at sub-micromolar concentration [141];
- 2-chloro benzothiophene derivative **12** and aza-benzimidazole derivative **13**, as two orally bioavailable DOT1L inhibitors [142];
- the phenoxyacetamide derivatives L01, L03, L04, and L05, identified by hierarchical docking-based virtual screening and molecular dynamic simulation [143];
- and massonianoside B, identified by use of a pharmacophore-based in silico screening [144].

#### 3.1.2. Targeting the Menin/Mixed Lineage Leukemia (MLL) Proteins Interaction Interface

The menin/LEDGF complex interaction with MLL is also crucial for MPAL and controls the expression of HOXA transcription factors. Development of menin/MLL inhibitors is another promising therapeutic strategy [145] (Figure 2, point **b**; Table 2). Most of those inhibitors are in preclinical evaluation, such as the macrocyclic peptidomimetic MCP-1. This is also the case of the thienopyrimidine MI-2-2 [146] and derivatives MI-463 and MI-503, which both interact with menin at the nanomolar range [147]. In a mouse model, MI-2-2 reduced by ~80% Hoxa9 and Meis1 expression, induced differentiation as evidenced by the presence of CD11b marker on treated cells, and presented a strong anti-clonogenic activity; however, it could not be evaluated in vivo due to poor stability [146]. By contrast, MI-463 and MI-503 are metabolically more stable than MI-2-2, are highly efficient in induction of the differentiation process in several AML cell models and evidence interesting in vivo anti-leukemic activities. MI-503 seems to be the most promising of those two derivatives because of deeper contacts within the menin pocket [147]. Its hydroxylated derivative MI-538 has also been selected for its even higher properties to optimize oral bioavailability with the hope to enter clinical trials [148]. Recently, the thienopyrimidine compound MI-1481 was also selected as a strong menin/MLL inhibitor (IC50 of 3.6 nM). MI-1481 reduced HOXA9 and MEIS1 expression and increased CD11b gene expression in a MV4-11 human AML cell model in culture, as well as in vivo, as shown in MV4-11 cells isolated from bone marrow and the spleen after six days’ treatment with MI-1481 of MV4-11 engrafted mice [148]. 

Another derivative, KO-539, has been developed by Kura oncology and is the most advanced menin inhibitor for AML therapy being proposed for phase I clinical trials in relapsed or refractory AML. KO-539 was identified using high-throughput virtual screening followed by structure-based optimization evidencing an IC50 of 22 nM for menin/MLL binding inhibition and a cellular activity at the nanomolar range on cell lines bearing MLL fusions with AF4, AF9, or ENL partners (either in AML or ALL cell models of MPAL), but at the micromolar range in non-MLL leukemic models. In animal models, KO-539 was given by the oral route and evidenced weak toxicity in mice and potent leukemic cells differentiation (CD11b-expressing cells in bone marrow of treated mice) and anti-leukemic effects (reduced splenomegaly and increased global survival) in both the MV4-11 MPAL model and patient-derived xenografts from the NPM1c+/FLT3-mut AML subtype [149]. Among other menin inhibitors are the hydroxyl-methyl-piperidines ML227, MIV-6, and their cyclopentyl-phenyl-piperidine derivative M-525, which all mimic the MLL-interacting peptide [150,151,152]. Such compounds were evaluated in combination with DOT1L inhibitors to restore differentiation in MPAL cell models [153]. However, the development of ML227 was impaired due to poor metabolic stability, relatively weak activity (IC50 for binding to menin is 390 nM) as well as off target activities [151]. Single change of the hydroxyl group of ML227 by an amine group to obtain MIV-6 led to a more stable compound but did not significantly increase the IC50 for menin recognition (~185 nM). By contrast, another structure/activity relationship study led to the selection of M-525 as a much more efficient compound to interact with menin protein (IC50~3.3 nM) with higher specificity and efficacy in MPAL cell models such as MV4-11 [152].

Interestingly, DOT1L collaborates also with the bromodomain protein BRD4 [154], an acetyl-histone H4 binding protein that represents another interesting target to treat AML [155]. Inhibitors of BRD4 were selected against different acute leukemia, including MPAL: I-BET762 (GSK525762), OTX015/MK8628 (Birabresib), CPI-0610, FT-1101, BI-894999, BMS-986158, PLX51107, RO6870810, and GSK2820151, among others, which entered clinical trials in different pathologies, [156] while other compounds, such as thienotriazolodiazepine (+)-JQ1, MS436, the iridium based inhibitor 1a or the 3-hydroxyisoindolin-1-one derivate 10e are at developmental stages [154,157,158,159,160,161,162,163] (Figure 2, point **c**; Table 2). However, some authors suggested that BRD4 inhibitors act independently of HOX genes [164]. Since the dimethylation of H3K79 induced by DOT1L favors the acetylation of H4 histone as an epigenetic mark recognized by BRD4, BRD4 may in this way participate in DOT1L function, and the concomitant use of both DOT1L and BRD4 inhibitors may synergistically inhibit proliferation of MLL-rearranged leukemic cells [154].

#### 3.1.3. Targeting WDR5/MLL Interaction

The interaction of WDR5 with MLL protein is the third approach presented here (Figure 2, point **d**; Table 2). Inhibitors of WDR5/MLL interaction were developed as peptidomimetics, such as MM-102, which interacts with crucial amino acids of WDR5 [165] for an inhibition constant Ki at sub-nanomolar range. MM-102 reduces HOXA9 and MEIS1 expression in a murine bone marrow cell model transformed by transduction of the MLL-AF9 fusion protein and induces cell death in the MV4-11 and KOPN-8 cells expressing MLL-AF4 and MLL-ENL, respectively, but not in the K562 cell line expressing wild-type MLL [166]. 

The macrocyclic MM-401 and MM-589 peptidomimetics are also high affinity WDR5 binding compounds. MM-401 induces modification of gene expression profile with common features with that of the depletion of MLL-fusion in the murine MLL-AF9 cell model, among which are the *hoxa9* and *hoxa10* gene expression level or genes associated with G1/S phase arrest and apoptosis. Cell cycle arrest, cell death and expression of the myeloid surface marker CD11b were also detected in the human MV4-11 cell model but not in the K562 cell line [167]. MM-589 is a strong inhibitor with Ki of 0.9 nM, that is specific to MLL1 but inactive in other MLL proteins. MM-589 is efficient in human AML cell lines with MLL-AF9 (MOLM-13) or MLL-AF4 (MV4-11) fusions but not on the HL-60 cell line with wild-type MLL [168].

In addition to peptidomimetics mostly having poor cellular permeability, small molecules were designed as antagonists. Among these is WDR5-0102, which binds the arginine pocket of WDR5 [169], and also its derivatives WDR5-47 [170], the pyridyl-derivative compound 23 [171], OICR-9429 [172] and finally DDO-2117, as the most active with binding constants at sub-nanomolar range [173,174].

#### 3.1.4. Targeting Cyclin-Dependent Kinase 9 (CDK9) 

The MLL-fusion proteins also interact with pTEFb, a transcription elongation factor complex that phosphorylates serine 2 residues located in the heptad repeats “YSPTSPS” from the carboxyl-terminal domain of the largest subunit of RNA-PolII, thus activating the transition from the initiation to the elongation step of the transcription process. Therefore, targeting pTEFb was evidenced as a strategy to treat MPAL (Figure 2, point **e**; Table 2). pTEFb is composed of two proteins: CDK9 and cyclin T1. Flavopiridol (alvocidib) was identified as a potent CDK9 inhibitor and the first-in-class CDK inhibitor that has entered clinical trials in AML [175,176], followed by BAY-1143572 [177], and the pan-CDK inhibitors dinaciclib [178] or TG02 [179].

Other CDK9 or pan-CDK inhibitors are in developmental stage, such as LS-007/CDKI-73 [180], LY2857785 [181] or, more recently, JSH-150, which inhibited CDK9 at the nM range and showed interesting anti-leukemic effects in mice inoculated with the MV4-11 human AML-AF4 MPAL model [182]. CDK9 inhibitors also synergize with BRD4 inhibitors such as BI-894999 [163]. However, besides the association of CDK9 with MLL-fusion proteins, none of the studies with CDK9 inhibitors evaluated HOXA9 gene expression.

#### 3.1.5. Targeting Mixed Lineage Leukemia (MLL) Protein Degradation

Recently, the inhibition of the degradation by the proteasome of the wild-type MLL was proposed as an alternative strategy against MPAL with the use of IRAK (interleukin-1 receptor-associated kinase) inhibitors (Figure 2, point **f**; Table 2). In response to IL1B (interleukin 1 beta), IRAK4 phosphorylates the E3-independent E2 ubiquitin-conjugating enzyme UBE2O protein that consequently interacts with the C-terminus part of wild-type MLL protein (a portion that is absent in MLL-fused proteins) to favor MLL protein degradation by the proteasome. Inhibition of wild-type MLL degradation by IRAK4 inhibitors may therefore increase the level of wild-type MLL protein that is thought to compete with the oncogenic MLL-fused proteins for binding to the DNA. Treatment of cells with the IRAK1/4 inhibitor for 24 hours increases wild-type MLL protein binding to the HOXA cluster and the survival of mice previously inoculated with MLL-AF9-transformed murine hematopoietic cells [122].

#### 3.1.6. Targeting Other Epigenetic Factors Associated with the MLL Complex

In MPAL, MLL translocations lead to the loss of methyltransferase function, resulting in the recruitment of other antagonist epigenetic modulators such as the histone lysine-specific demethylase 1 (LSD1/ KDM1A) (Figure 2, point **g**; Table 2). LSD1 demethylates H3K4me1/2 and H3K9me1/2, leading to the repression of the transcription of genes responsible for differentiation blockade [123,183]. LSD1 knockdown [184] or inhibition by the irreversible inhibitor GSK-LSD1 [185] leads to the differentiation in Hoxa9/Meis1 positive murine AML cells. Consequently, LSD1 inhibitors activate the differentiation cascade and regulate the cell cycle process [186]. As an example, Feng et al. [186] synthesized cyclopropylamine-based compounds that are active at the nanomolar range on the MPAL cell models MV4-11 and MOLM-13, reducing HOXA9 and MEIS1 gene expression and inducing the expression of CD11b and CD14 differentiation markers at the surface of the cell, arguing for an active myeloid differentiation process. This mode of action is observed in MLL-fusion models but LSD1 inhibitors are much less active (µM range) in non-MLL cell models, even those over-expressing HOXA9, such as the U937 AML cell model (CALM-AF10 fusion).

The most promising LSD1 inhibitors in AML appear to be (i) the tranylcypromine-based compounds such as tranylcypromine (TCP) trentinoin (in combination with all-trans retinoic acid, ATRA) against relapsed/refractory AMLs, (ii) GSK2879552 (GlaxoSmithKline), evaluated in small cell lung cancer and AML, and (iii) ORY-1001 (iadademstat, Oryzon Genomics), which entered phase I clinical trials in AML [187,188]. Those compounds act as irreversible inhibitors of KDM1A. It was evidenced that GSK2879552 treatment reduces HOXA9 expression and cell viability in the MOLM-13 MPAL cell line [189] and that ORY-1001 treatment of THP-1 MPAL cell model down-regulates HOXA9/10/11 gene expression, induces differentiation and modifies the epigenetic marks of various differentiation genes, such as CD11b, S100A12, and LY96, resulting in their over-expression, and finally presents good anti-leukemic activities in mice [190].

In addition to those compounds that have entered clinic trials, others are also being evaluated in pre-clinical models. The LSD1 antagonist SP2509 was active in the MPAL cell model MOLM-13, but also in the NPM1c+ cell model OCI-AML3, inducing cell differentiation, but the effect on HOXA9 expression was not evaluated. SP2509 evidenced promising results used in combination with the HDAC inhibitor panobinostat in OCI-AML3 and patient-derived xenograft mice models [191]. NCD38, a tranylcypromine-based LSD1 inhibitor that impairs growth of the MLL-AF9-positive leukemia cell model MOLM14, reduces HOXA9 expression and induces myeloid differentiation [192]. JL1037, selected by computational screening, evidenced G0/G1 cell arrest in the THP-1 cell model (MLL-AF9) [193] or the 4-cyanophenyl-glycine derivative **32** active at the nanomolar range on LSD1 as evidenced by surface plasmon resonance [194].

The epigenetic writer H4R3 methyltransferase PRMT1 and the eraser Jumonji domain-containing H3K9 demethylase KDM4C (JMJD2C/GASC1) are two proteins that also associate with the oncogenic MLL complex, as evidenced in MLL-AF9 and MLL-GAS7 translocation models [195]. Those two epigenetic factors could be inhibited by small compounds, namely, AMI-408 [196] and the *N1*-(2-((2-chlorophenyl)thio)benzyl)-*N1*-methylethane -1,2-diamine compound (**28d**, DCPR049_12) [197] against PRMT1 (Figure 2, point **h**), and SD70 [198] against KDM4C (Figure 2, point **i**; Table 2). In particular, **28d** was able to reduce HOXA9 and MEIS1 expression in MPAL models [197].

The H4K16 histone acetyl-transferase (HAT) MOF is another epigenetic writer associated with MLL-AF9- and NUP98-HOXA9-driven leukemias. The MOF inhibitor MG149 evidenced anti-leukemic activities of MLL-fused leukemia and other AML cell models that are associated with HOXA9 over-expression, such as the CALM-AF10 fusion model U937, but also the K562 cell model (BCR-ABL fusion) that does not express HOXA9 [199], suggesting a more global epigenetic deregulation by MOF inhibitors that is not restricted to deregulated HOXA9 gene expression (Figure 2, point **j**; Table 2).

### 3.2. Targeting HOXA9 Expression Through MLL-Independent Epigenetic Modifiers

Targeting epigenetic modifiers could also prevent HOXA9 transcription in other AML sub-types. The activity of these epigenetic enzymes is modulated, for instance, through metabolites produced by mitochondrial metabolism.

The dihydroorotate dehydrogenase enzyme (DHODH) is involved in nucleic acid synthesis and the cell cycle, as well as in the post-translational glycosylation of important protein targets such as epigenetic enzymes [200]. The inhibition of DHODH leads to the depletion of pyrimidine precursors and inhibition of nucleic acid synthesis enabling myeloid differentiation in human AML cells and in a mouse AML model expressing an estrogen-dependent form of Hoxa9. Multiple DHODH inhibitors were evaluated in AML or other pathologies, including malaria and fungi infections [201,202]. In vivo, DHODH inhibitors reduced leukemic cell burden, decreased levels of leukemia-initiating cells, and improved survival [203] (Figure 2, point **k**; Table 2).

Both IDH1 and IDH2 proteins are glycolytic enzymes that catalyzes the conversion of isocitrate into α-ketoglutarate. IDH1/2 mutations are found in ~15%–20% of secondary and de novo AML and globally impact the normal catalytic activity by leading to the production of 2-hydroxyglutarate (D-2-HG). This oncometabolite is a competitive inhibitor of the epigenetic TET2 enzyme, thus leading to the decrease of 5hmc marks [204,205]. It was shown that the cooperation of IDH1 or IDH2 and NPM1 mutations are associated with the activation of HOXA9 expression [50]. Furthermore, the mutant IDH1 cooperates with Hoxa9 to greatly accelerate myeloproliferative disease-like myeloid leukemia in mice [96]. The first agent developed against IDH2 mutated enzyme is enasidenib (also known previously as AG221), which induces differentiation of leukemic cells [206]. Enasidenib (IDHIFA, Celgene Corp.) was approved in 2017 for the treatment of relapsed or refractory AMLs (Figure 2, point **l**; Table 2). The first clinical trials evidenced promising results but patients unfortunately developed high differentiation syndrome, as described for ATRA treatment in APL [207]. Then, in 2018, the U.S. Food and Drug Administration (FDA) approved ivosidenib (Tibsovo, Agios Pharmaceuticals, Inc., Cambridge, USA) as the first-in-class IDH1 inhibitor for patients with relapsed or refractory AML presenting an IDH1 mutation. Ivosidenib was approved simultaneously with the RealTime IDH1 Assay, a companion diagnostic that is used to quickly detect IDH1 mutations. New IDH-mutated enzymes inhibitors are currently being developed and clinical trials combining enasidenib and hypomethylating agents are currently being evaluated [208].

At the level of epigenetically modified DNA, different mutations of the DNA methyl transferase protein DNMT3A are associated with modifications of the DNA methylation status, leading to a leukemia associated with HOXA9/MEIS1 expression [209,210,211]. The most frequent DNMT3A mutation is R882H (>80% of all mutated DNMT3A). Two azanucleotide DNMT inhibitors, azacitidine and decitabine, are currently used in AML treatment (Figure 2, point **m**; Table 2). In patients with DNMT3A gene mutations associated with worse outcomes and HOXA9 over-expression, decitabine treatment is associated with higher levels of complete remission in comparison with AML patients having wild-type DNMT3A, suggesting that AML patients with low DNMT3A activity due to those loss-of-function mutations benefit from treatment with hypomethylating agents such as decitabine [212]. DNMT3A-mutated proteins interact with different proteins, such as the base excision repair enzyme thymine DNA glycosylase (TDG) or/and the DNMT3-like protein, in a manner that changes upon the point mutation localization [211]. This may represent two new original ways to develop protein/protein inhibitors for AML treatment in the future.

At the histone level, HDAC inhibitors such as valproic acid (Depakote^®^, Sanofi Aventis, Gentilly, France), vorinostat (suberoylanilide hydroxamic acid, SAHA, Zolinza^®^, Merck & Co., Inc., USA), the cinnamic hydroxamic acid analogue panobinostat (LBH589, Farydak^®^, Novartis Pharmaceuticals, East Hanover, USA), romidepsin (Istodax^®^, Celgene corporation, Summit, NJ, USA), belinostat (PXD101, Beleodaq^®^, Spectrum Pharmaceuticals, Inc, Irvine, CA, USA), the phenylbutyrate-derivative AR-42 [213], the benzylcarbamate derivative entinostat (MS-275) [214], or mocetinostat could be an alternative to regulate gene expression (among HOXs) [189] and have been evaluated in clinical trials against AML, mainly in combination with other drugs [215,216,217,218] (Figure 2, point **n**; Table 2). The zinc finger transcriptional repressor GFI1 also regulates HOXA9 expression through recruitment of epigenetic factors, including HDAC, LSD1, and G9a. It is worth noting that a single nucleotide polymorphism (SNP) variant GFI1-S36N, present in ~5% of the Caucasian population, resulting in an elevated rapidity in the evolution of myelodysplastic syndrome to become AML, is associated with reduced epigenetic control at the Hoxa9 locus in a mouse leukemia model. Treatment with HDAC inhibitors would therefore help to control the expression of HOXA9 in this GFI1-S36N SNP population [97].

Targeting NPM1c+ is another means of inhibiting HOXA9 expression. Wild-type NPM1 (also called B23 or numatrin) is a nucleolar phosphoprotein that preferentially binds to quadruplex DNA, a function and localization that is lost in NPM1c+; inhibitors of NPM1 have been developed [219]. The retinoid deguelin was the first identified as an inhibitor of NPM1c+ expression by inducing an increase in the proteasome degradation of NPM1c+, but not of wild-type NPM1, through a yet unknown mechanism. As a consequence, deguelin induces both cell death and cell differentiation in the NPM1c+ AML cell line model OCI-AML3 [220,221]. Interestingly, deguelin has multiple mechanisms of action in AML and also increases NUP98 binding to the nuclear pore in the U937 cell line [222]; possible re-localization of NUP98-HOXA9 from DNA to the nuclear pore under deguelin treatment, and the subsequent consequences in terms of cell survival and differentiation, would be a mechanism that would be interesting to evaluate. In parallel, wild-type NPM1 inhibitors were designed to block NPM1 oligomerization, such as NSC348884, which binds at the dimer interface of two NPM1 proteins to abolish NPM1 dimerization [223]. NSC348884 was evaluated in the NPM1c+ AML cell model and patient samples [224]. In 2017, the imidazoquinoxaline derivative EAPB0503 was found as a NPM1c+ inhibition active in AML and reduced cell growth in the NPM1c+ OCI-AML3 cell line and patient samples, but not in wild-type NPM1 THP-1 and MOLM-13 cell lines or wild-type NPM1 patient samples. Interestingly, EAPB0503 also evidenced valuable anti-leukemic effects in vivo on OCI-AML3, but not THP-1, engrafted mice [225]. However, the impact on HOXA9 expression, associated with NPM1c+ expression, has not yet been evaluated for any of these inhibitors (Figure 2, point **o**; Table 2).

## 4. Direct Targeting of HOXA9

As presented in the previous section, indirect inhibition of HOXA9 leading to the repression of its expression could be achieved through a broad diversity of transcription regulators used as targets, and related drug candidates.

Direct targeting of HOXA9 protein is undoubtedly an interesting strategy to treat a large proportion of AML. As a transcription factor, HOXA9 does not belong to receptor or steroid families that could be targeted by ligand mimetics through the binding to a binding pocket. Two other strategies are currently being developed: targeting HOXA9 at the transcription factor/cofactor protein/protein interface and targeting HOXA9 at the DNA binding level.

### 4.1. Direct Targeting of HOXA9 Using Inhibitors of HOXA9/Cofactor Interaction

**Figure 3:**
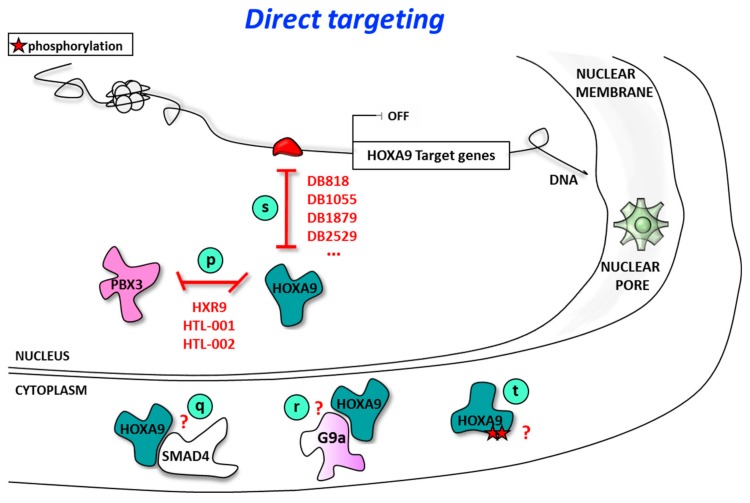
Direct targeting of HOXA9 function: inhibition of protein/protein or protein/DNA interaction to block HOXA9 transcriptional activity in AML.

Targeting the protein/protein interface is one strategy that has already been developed to target the HOX protein though its interaction with specific co-factors (Figure 3), since the interaction of HOXA9 with some cofactors is a key element for leukemia induction and aggressiveness [29]. PBX3 is a critical co-factor of HOXA9 in leukemogenesis [100]. The interaction of PBX co-factors (PBX1 to 4) with HOX proteins confers both an increase in the DNA binding affinity and an enlarged and more selective DNA binding site [226,227,228]. PBX proteins interact with proteins from HOX1 to 11 paralog genes though a highly conserved hexapeptide with crucial YPWM amino acids [10,226,229,230], resulting in a conformation change upon cooperative binding with their DNA cognate sequence [231,232,233,234]. Information from this HOX/PBX/DNA complex structure was the template for the development of HOX/PBX protein/protein inhibitors. Very recently, Merabet and co-workers used in vitro DNA binding studies and cellular bimolecular fluorescence complementation (BiFC) experiments using a series of mutants to evidence that HOXA9 binding to PBX1 requires the hexapeptide in addition to two paralog-specific residues within HOXA9, HOXB9, and HOXC9 homeodomains: an arginine residue at position 234 for human HOXA9, located between helix 1 and helix 2, and a methionine residue at position 260 for human HOXA9 within the helix 3 associated with direct DNA recognition [235].

The peptide inhibitor HXR9 (Figure 3, point p) was developed more than a decade ago with the view to target the HOX/PBX protein/protein interaction surface [236] and is composed of 18 amino-acids with the hexapeptide sequence (YPWM) and a repeat of nine arginine residues to facilitate cell delivery: WYPWMKKHHRRRRRRRRR. HXR9 disrupts the interaction of the hexapeptide sequence from multiple HOX proteins, which is used to interact with different PBX partners. HXR9 binds to the pocket in PBX, which otherwise would be recognized by HOX proteins. Such interaction shuts down the expression of various genes involved in apoptosis [8,237,238]. Disruption of HOX/PBX interaction impacts cell proliferation and restores cell death in various solid tumor cell models [238], including melanoma [236,239], renal cell carcinoma [240], ovarian cancer (such as the SK-OV3 cell model or primary samples [241,242]), breast cancer [243], prostate cancer [244], asbestos-associated mesothelioma [245], and oral and esophageal squamous cell carcinoma [246,247]. HXR9 was also evaluated in AMLs based on the relevance of HOXA9 and PBX3 interaction in AML [29,75,248,249], but other expressed HOX/PBX3 interfaces may also be disrupted by HXR9 in AML. In mouse cell models, HXR9 affects cell survival of murine hematopoietic progenitor cells transduced with MLL-AF9 or with both HOXA9 and PBX3. Interestingly, HXR9 reduced cell viability in a variety of HOXA7/9/11-positive cell line and blasts from patients but not on HOXA7/9/11-negative ones. HXR9 treatment also reduced the expression of known HOXA9 target genes, including MEF2C, MYB, and FLT3 in human HOXA9-positive cell lines but not in negative ones [8,248]. HXR9 also induces an increase in cell surface presentation of differentiation markers CD11b and/or CD14 in THP-1/OCI-AML3 human cell models. In the human MLL-AF9-expressing MPAL cell model MONOMAC-6, HXR9 treatment induced an inhibition of cell growth and cell viability (IC_50_ around 10 µM), associated with an increase in apoptotic cell death, as evidenced by flow cytometry after propidium iodide/annexin V double staining. Of interest, HXR9 is also active in the HOXA9-negative cell line K562, an erythroleukemia cell line derived from a chronic myeloid leukemia patient in blast crisis and associated with BCR-ABL fusion kinase that may express other HOX proteins.

The HXR9 derivative peptide HTL-001 (Figure 3, point p) (WYKWMKKAARRRRRRRRR; underlined are the modified amino acids relative to HXR9) also effective against numerous cell lines and human tumors, and is also licensed by HOX Therapeutics Ltd. (HTL, Guildford, UK). Among them are the PC3 prostate and MDA-MB-231 breast cancer models against which the peptide inhibitor HTL-001 presented higher efficacy that the parental HXR9 peptide [250]. Given that HTL-001 doubles the global survival of mice developing glioma and that HTL-001 is well tolerated in mice, rats, and rabbits, a phase I clinical trial involving around 20 patients treated with intravenous HTL-001 is planned at the end of 2019 in patients with multiform glioblastoma, a particularly aggressive and adverse cancer.

More recently, HOX Therapeutics Ltd. developed a small molecule, HTL-002 (Figure 3, point p) as a potential orally available pharmacological inhibitor of HOX/PBX interaction based on the HTL-001 structure, which might be another promising way to inhibit HOX/PBX in various cancers, however, no publications are yet available.

The PBX and MEIS proteins are not the only HOXA9-interacting proteins. For instance, SMAD4 (Mothers against decapentaplegic homolog 4), a TGF-β and bone morphogenic protein (BMP) signaling pathways associated factor, interacts with HOXA9 (Figure 3, point q) as demonstrated by co-immunoprecipitation. SMAD4 interferes with HOXA9 binding to the osteopontin (OPN) gene promoter through which HOXA9 represses OPN expression [251]. By contrast with the PBX/HOX protein/protein interaction that stabilized the binding to their DNA cognate sequence, the SMAD/HOX protein/protein interaction occurs through the MH1 domains of SMAD proteins in a manner that abolishes HOXA9 binding to DNA [252,253,254]. Indeed, through interaction with SMAD4, HOXA9 (as well as the oncogenic fusion protein NUP98-HOXA9) was removed from DNA [253] and sequestrated in the cytoplasm to protect normal hematopoietic stem cells and progenitor cell (HSPC) transformation by HOXA9 or NUP98-HOXA9, as evidenced in a mouse model of AML [254]. Consequently, increasing SMAD4 expression or stabilizing the SMAD4/HOXA9 complex by taking advantage of the structural properties of their interaction [255] would be another interesting opportunity to target HOXA9 leukemogenic function.

The histone H3 lysine 9 methyltransferase G9a (KMT1C/EHMT2) is also known to physically interact with HOXA9 in AML as determined by co-immunoprecipitation [256] (Figure 3, point r). Inhibition of G9a using UNC0648 in murine Hoxa9/Meis1A-transformed cells inhibits cell growth, induces myeloid differentiation, and correlates with the deregulation of HOXA9 target genes as exemplified by gene expression correlations using GSEA software in this murine model [256]. However, such myeloid differentiation by UNC0648 was not observed by other authors on three evaluated AML cell lines. By contrast, the peptide-competitive inhibitor A-366 evidenced some leukemic cell differentiation properties as shown by flow cytometry on CD11b myeloid differentiation marker in the MV4-11 MPAL cell model [257]. However, it is yet unclear if the mechanism of action of UNC0648 and other G9a inhibitors that bind to the peptide substrate pocket of G9a is reliable for G9a/HOXA9 interaction or, more likely, for epigenetic control (Figure 2, point r).

### 4.2. Direct Targeting of HOXA9/DNA Interaction Using Sequence-Selective DNA Ligands

Protein/DNA is another interface that could be targeted to inhibit a transcription factor. This could be achieved through binding to the DNA binding domain when a binding pocket is present or identified or, more commonly, through binding to their DNA cognate sequence [2,258]. Inhibitors that directly recognize some DNA binding domains are already identified in different classes of DNA binding domains, such as hormone receptors (PRIMA-1 and derivatives interacting with mutated p53 oncogene [259,260]; VPC-14428 and VPC-14449 for binding to a pocket of the androgen receptor [261]; InS3-54 directly interacting with STAT3 DNA binding domain [262]; GANT61 targeting GLI1 and GLI2 DNA binding domains [263]; or BRD32048 against the ETS transcription factor member ETV1 [264]), but none currently target a homeobox-containing transcription factor.

The alternative to target transcription factor/DNA binding is to interfere at the level of DNA interaction. Such interference was already achieved using well-known alkylating drugs with either trapping of the protein/adduct or inhibition of the transcription factor/DNA interaction (cisplatin, trabectedin, and CC-1065, among others) [57,265,266,267,268,269]. The effect of such alkylating drugs on transcription factor/DNA binding as a therapeutic approach is of course biased by the effect of DNA alkylation per se, and the development of such compounds as pure transcription factor inhibitors is compromised. Non-alkylating drugs have also been developed, some intercalating between base pairs DNA intercalators, such as echinomycin inhibiting HIF1/DNA binding [270], MLN944 against c-JUN/DNA binding [271], or flavopiridol interfering with STAT3/DNA binding [272]. Besides alkylating drugs, other inhibitors bind in the DNA groove to make deep contacts with a larger DNA sequence. Among the latter transcription factor inhibitors are mithramycin and derivatives evaluated in clinical trials for their inhibition of SP1 or EWS-FLI1 binding to DNA [76,273,274,275,276,277,278,279,280], or pyrrole-imidazole polyamides recognizing a large variety of transcription factor cognate sequences [281,282,283]. A polyamide-peptide conjugate was designed to trap the drosophila homeodomain-containing protein extradenticle Exd to the DNA by mixing a polyamide as a minor groove DNA binding moiety to a peptidic moiety containing the YPWM HOX/PBX interaction peptide [284,285], but no currently synthesized polyamide is known to destabilize HOXA9/DNA interaction. Only one paper referred, more than 10 years ago, to a lactam carboxamide that was able to inhibit HOXA13/DNA binding [286].

Recently, we selected a series of heterocyclic diamidines as minor groove DNA ligands on the HOXA9 cognate sequence (Figure 3, point s). Among them, the diamidines derivatives DB818, DB1055, DB1879, and DB2529, which proved to be highly efficient competitors of the HOXA9/DNA interaction. The HOXA9/DNA binding inhibition occurs through a strong binding of the compounds within the minor groove of the HOXA9 cognate sequence. In particular, the diamidine phenyl-thiophene-benzimidazole DB818 and the diamidine diphenyl-benzimidazole DB1055 alter HOXA9-mediated transcription in luciferase assays. Both DB818 and DB1055 decrease cell survival but also increase cell death. Furthermore, granulocyte/monocyte differentiation was evidenced upon treatment with DB818 or DB1055 by a decrease in the number of colonies from more immature granulocyte-monocyte progenitor (CFU-GM) sub-types but to an increase of those corresponding to more differentiated ones from the granulocyte (CFU-G) or monocyte (CFU-M) compartments. The induction of cell death and hematopoietic cell differentiation were also highlighted using transcriptomic analysis of DB818-treated murine Hoxa9-transformed hematopoietic cells. Similarly, GSEA analyses of the DB818-treated murine Hoxa9-transformed cell line evidenced similarities between DB818-induced up-regulated genes and genes down-regulated upon NUP98-HOXA9 transformation, as well as between DB818-induced down-regulated genes and alteration of HOXA9 functions.

Overall, these data demonstrate for the first time the propensity of sequence-selective DNA ligands to inhibit HOXA9/DNA binding both in vitro and in a murine Hoxa9-dependent leukemic cell model [287]. Evaluation of DB818 and DB1055 on human AML cell lines and blasts isolated from AML patients evidences interesting differentiation and anti-leukemic activities, highlighting the targeting of the HOXA9 cognate sequence, and an original and promising strategy to inhibit HOXA9 leukemogenic function (submitted manuscript, personal communication).

In summary, the different approaches presented in this manuscript are gathered in Table 2.

## 5. Conclusions

We showed in this review that the physical (indirect) and functional (direct) inhibition of HOXA9 in AML could be achieved through multiple strategies, some being used/evaluated in clinic, and many others being in preclinical stage. For instance, indirect targeting strategies for epigenetic complex actors or cofactors are currently under development and in clinical trials, as illustrated by DOT1L inhibitors. MLL abnormalities, which are found in about ~5% of AML, are associated with a poor patient prognosis, justifying the strong need for new therapies against MPAL [153,295]. However, in parallel to its recruitment by the MLL complex deregulated in AML, DOT1L is also involved in the regulation of the cell cycle, as well as in the response to DNA damage [296]. Therefore, inhibition of DOT1L may potentially present some collateral effects, such as chromosomal instabilities and defects of normal hematopoiesis, which are reported for some DOT1L inhibitors [297,298].

Moreover, besides being an attractive strategy to control HOXA9 expression at the epigenetic level, it is worth noting that most, if not all, of the epigenetic drivers presented here have an effect on the expression of many other genes. Consequently, such approaches that change the epigenetic marks may not exclusively be active on the leukemic cells of AML patients, but may also present side-effects and off-target effects by altering normal cells [299,300,301,302]. Moreover, such epigenetic targeting would not be restricted to HOXA9 but be also active on other members of the posterior HOXA locus, namely HOXA5 to HOXA11, which are usually epigenetically co-expressed.

Based on actual knowledge, new therapeutic opportunities may also come from other aspects of the regulation of HOXA protein expression (e.g., CTCF, miRNA, LncRNA, and mRNA stability) or activity (e.g., other protein/protein interaction, phosphorylation, and degradation). Indeed, the binding of CCCTC-binding factor (CTCF) at chromatin loop bases around the HOX locus is associated with the silencing of HOX cluster expression with (CTCF) binding sites located at the loop bases, subsequently stabilizing the polycomb repressive complex 2 (PRC2) binding to DNA, and resulting in H3K27me3 repressive epigenetic mark trimethylated lysine 27 of histone H3 to lock the HOXA cluster [303,304]. In MPAL, the disruption by CRISPR-Cas9 of a CTCF binding site located between HOXA7/HOXA9 genes identified this site as a critical regulator of HOXA9 expression [305]. Similar approaches may be proposed against miRNAs or LncRNAs that are known to control HOXA9/PBX/MEIS expression in AML (such as miR-196b [306,307], miR-181 [248], let7c [307], miR-495 [308], HOTTIP [309]) or to control MLL and epigenetic drivers (such as miR-29b [310] and miR-9 [311]). The phosphorylation of HOXA9 by the serine-threonine kinases PKC or casein kinase II (CKII) is another direct way to inhibit HOXA9 and other HOX protein functions. Indeed, HOXA9 is phosphorylated by CKII on the S175 amino acid located outside of the homeodomain and by PKC on the S204 and T205 residues within the consensus phosphorylation site STRK located in a conserved region of its homeodomain. In particular, phosphorylation of the S204 residue by PKC strongly decreased HOXA9/DNA and HOXA9/PBX1/DNA complex formation in vitro, and PKC inhibition by bisindolyl-maleamide 1 decreases TPA-induced differentiation in a HOXA9-transformed murine cell line [107] (Figure 3, point t). Many other HOX proteins are also subjected to phosphorylation by PKC, CKII, cyclin-dependent kinase (CDK), MAPK, ATM/ATR, and other kinases as recently reviewed [312], but the precise function of many is still unclear and no therapeutic approaches have yet been developed in clinical trials, even if it has been shown that PKC inhibition by Ro31 enhances the effect of HXR9 peptide on necroptosis [8].

The observation that regulation at the expression or phosphorylation level would not be restricted to HOXA9 but be also effective on other HOX members could somewhat be made for the targeting of the HOX/PBX interface. Indeed, a large number of HOX proteins interact with PBX and their interaction is blocked using peptide inhibitors such as HXR9. It is not yet totally clear whether the co-expressed HOXA have redundancy that are essential or not to the AML pathology. Moreover, HOXA proteins present on the same locus, or paralog HOX proteins (HOX9 for instance) share a closely related HOX/PBX interaction interface, and some similarities in the DNA binding sequence and a better knowledge of their respective mechanisms of action would be determinant.

Identifying and targeting new HOXA9/protein interactions is another alternative research area of interest. At present, two non-PBX/MEIS proteins were identified as potential protein partners: SMAD4 and G9a. Identification of the precise protein/protein interfaces and subsequent drug screening may identify new potential drugs to treat AML.

Finally, there is also still some limitation in the targeting of the HOXA9/DNA interface, but a better knowledge of HOXA9 target genes and the way HOXA9 binds to the genome from comparison with the other 38 HOX proteins would help develop new strategies. Indeed, for the DNA binding inhibition strategy, the precise knowledge of the HOX specificity on the genome of a cancer cell is not fully addressed. This is mainly due to the absence of ChIP-seq analyses for each HOX protein expressed in a specific cell (for instance, AML here). AML is a nice model to compare HOXA regulated genes (using RNase-seq, for instance), binding sequences (using ChIP-seq as examples) and protein partners (using immunoprecipitation-coupled mass spectrometry of endogenous protein (RIME) or bimolecular fluorescence complementation (BiFC), for instance). However, the ChIP-seq and RIME approaches may be properly performed only by use of highly specific and efficient antibodies, validated against each HOX protein, and those antibodies are unfortunately not yet available. Such kinds of global approaches, compiled with other high throughput and next-generation sequencing analyses (e.g., DNase-Seq, SELEX-seq, ChIA-Pet, 4C-seq, cleavage under targets and release using nuclease (CUT&RUN) analyses, methylome, ATAC-seq, and binding energy topography by sequencing (BET-seq), etc.) would also provide opportunities to identify critical downstream factors as new potential targets in AML and other cancers associated with HOXA9 over-expression.

## Figures and Tables

**Table 1:** List of the main genetic alterations in AML that are associated with HOXA9 over-expression.

Type of Alteration	Fusion/Mutation/Additional Chromosome	Translocation/Inversion/Deletion	References
Chromosomal alterations	MLL fusions	11q23 translocations	[43,45,47]
	NUP98-NSD1	t(5;11)(q35;p15)	[80,82]
	NUP98-HOXA9	t(7;11)(p15;p15)	[83]
	NUP98-HOXA10	t(7;11)(p15;p15)	[84]
	NUP98-HOXC11	t(11;12)(p15;q13)	[85]
	NUP98-HOXD11	t(2;11)(q31;p15)	[86]
	NUP98-HOXD13	t(2;11)(q31;p15)	[84]
	NUP98-HHEX	t(10;11)(q23;p15)	[87]
	NUP98-KDM5A	t(11;12)(p15;p13)	[33]
	NUP98-PHF23	t(11;17)(p15;p13)	[33]
	NUP98-PRRX1	t(1;11)(q24;p15)	[33]
	NUP98-DDX10	inv(11)(p15q22)	[33]
	MYST3-CREBBP	t(8;16)(p11;p13)	[88]
	RUNX1-EVI1	t(3;21)(q26;q22)	[89]
	CDX2-ETV6	t(12;13)(p13;q12)	[90]
	CALM-AF10	t(10;11)(p12-14;q14-21)	[91]
	SET-NUP214	del(9)(q34.11;q34.13)	[92]
	NPM1-MLF1	t(3;5)(q25;q34)	[93,94]
	+8	/	[81]
Mutations	NPM1		[48,49,50,75]
	MLL-PTD		[42]
	DNMT3A		[95]
	EZH2		[42]
	IDH1/2		[50,96]
Polymorphism	GFI1-S36N		[97]

**Table 2:** List of therapeutic approaches and developed inhibitors for indirect or direct targeting of HOXA9 presented in this review (Cpd, compound, in bold: inhibitors used in clinic or that entered clinical trials).

Type of Targeting		Target		Inhibitor	Stage of development	References
Indirect Targeting of HOXA9	Epigenetic Control of HOXA9 expression	Proteins interaction within the MLL Complex	DOT1L	EPZ-5676	Phase I	[128,129,130,131,132,133,134]
				EPZ004777	Preclinical Stage	[129,130,131,132,133]
				SYC-522		[138]
				Cpd 9e		[139]
				Cpd 3, 9		[140]
				DC_L115		[141]
				Cpd 12, 13		[142]
				L01, L03, L04, L05		[143]
				Massonianoside B		[144]
			Menin/ LEDGF	KO-539	Preclinical Stage	[149]
				MCP-1		[147]
				MI-2-2		[146]
				MI-463		[147]
				MI-503		[147]
				MI-538		[148]
				MI-1481		[148]
				ML227		[151]
				MIV-6		[152]
				M-525		[152]
			BRD4	I-BET762	Phase I/II	[156,157]
				OTX015	Phase I/Ib	
				CPI-0610	Phase I/II	
				FT-1101	Phase I	
				BI-894999	Phase I	
				BMS-986158	Phase I/II (2019)	
				PLX51107	Phase I	
Indirect Targeting of HOXA9	Epigenetic Control of HOXA9 expression	Proteins interaction within the MLL Complex (continued)	BRD4	RO6870810	Phase I	[156,157]
				GSK2820151	Phase I	
				(+)-JQ	Preclinical Stage	[159,160,161,162,163]
				MS436		
				Ir-based cpd1a		
				Isoindoline derivate 10e		[161]
			WDR5	MM-102	Preclinical Stage	[166]
				MM-401		[167]
				MM-589		[168]
				WDR5-0102		[169]
				WDR5-47		[170]
				Cpd 23		[171]
				OICR-9429		[172]
				DDO-2117		[173]
			CDK9	Flavopiridol	Phase I/II	[175,176]
				BAY-1143572	Phase I	[177]
				Dinaciclib	Phase I/II/III	[178]
				TG02	Phase I	[179]
				LS-007	Preclinical Stage	[180]
				LY2857785		[181]
				JSH-150		[182]
		Other epigenetic modulators	LSD1	Tranylcypromine (TCP)	Phase I	[187,188]
				GSK2879552		[187,188]
				ORY-1001		[188,190]
				Cyclopropyl cpds	Preclinical Stage	[186]
				SP2509		[191]
				NCD38		[192]
				JL1037		[193]
				Cpd 32		[194]
			PRMT1	AMI-408	Preclinical Stage	[196]
				DCPR049_12		[197]
			KDM4C	SD70	Preclinical Stage	[198]
			MOF	MG149	Preclinical Stage	[199]
			DNMT3A	Azacitidine	FDA approval in AML	[212]
				Decitabine		
			HDAC	Valproic acid	Phase I/II in AML	[213,214,215,216,217,218]
				SAHA		
				LBH589		
				Romidepsin		
				Belinostat		
				Entinostat		
				Mocetinostat		
				AR-42		
			G9a	UNC0648	Preclinical Stage	[256]
				A-366		[257]
		Metabolism proteins	DHODH	Brequinar	Phase II	[288]
				BAY2402234	Phase I	[289]
				ASLAN003	Phase II	[290]
				ML390	Preclinical Stage	[291]
				NSC665564		[292]
				Isobavachalcone		[293]
				PTC299	Preclinical Stage	[294]
			IDH1	Ivosidenib	FDA approval in AML	[207,208]
			IDH2	Enasidenib	FDA approval in AML	[206]
	Other regulators of HOXA9 expression		NPM1c+	Deguelin	Preclinical Stage	[222]
				NSC348884		[223,224]
				EAPB0503		[225]
	Degradation		MLL	IRAK4 inhibitor	Preclinical Stage	[122]
Direct HOXA9 Targeting	Protein/protein interaction		HOX/PBX interaction	HXR9	Phase I	[70,75,248,249]
				HTL-001	Phase I (end 2019)	[250]
				HTL-002	Preclinical Stage	[HOX Therapeutics Ltd.]
	Protein/ DNA interaction		HOXA9 DNA binding site	DB818	Preclinical Stage	[287]
				DB1055		
				DB1879		
				DB2529		

## Abbreviations

AML	acute myeloid leukemia
ATAC-seq	assay for transposase-accessible chromatin with high throughput sequencing
ATM	ataxia telangiectasia mutated
ATR	ATM and RAD3-related
ATRA	all-trans retinoic acid
BET-seq	binding energy topography by sequencing
BiFC	bimolecular fluorescence complementation
BMP	bone morphogenic protein
BRD4	bromodomain-related protein 4
CBP	CREB-binding protein
CDK9	cyclin-dependent kinase 9
CFU-G	colony forming unit-granulocyte
CFU-GM	colony forming unit-granulocyte and monocyte
CFU-M	colony forming unit-monocyte
ChIA-Pet	chromatin interaction analysis by paired-end tag
ChIP	chromatine immune-precipitation CKII, casein kinase II
CTCF	CCCTC-binding factor
CUT&RUN	cleavage under targets and release using nuclease
D-2-HG	D-2-hydroxyglutarate
DHODH	dihydroorotate dehydrogenase
DNase-Seq	deoxyribonuclease I (DNase I)-hypersensitive site with high throughput sequencing
DNMT	DNA methyl transferase
DOT1L	disruptor of telomeric silencing 1-like protein
Erg	ETS-related gene
EVI1	ecotopic viral integration site 1
FLT3	Fms-like tyrosine kinase 3
GFI1	growth factor independent 1
GSEA	gene set enrichment analysis
HAT	histone acetyl-transferase
HD	homeodomain
HDAC	histone deacetylase
HEXIM1	hexamethylene bisacetamide (HMBA) inducible protein 1
HMT	histone methyltransferase
HOXA9	homeobox A9
IDH	isocitrate dehydrogenase
IL1B	interleukin 1 beta
IRAK4	interleukin-1 receptor-associated kinase 4
KDM4C	lysine-specific demethylase 4C
LEDGF	lens epithelium-derived growth factor
Lmo2	LIM domain only 2
LSD1	lysine-specific demethylase 1
MAPK	mitogen-activated protein kinase
MEF2C	myocyte enhancer factor 2C
MEIS	myeloid ecotropic viral integration site
MLL	mixed lineage leukemia
MOF	males absent on the first
MPAL	mixed-phenotype associated lineage leukemia
Myb	myeloblastosis gene
NPM1	nucleophosmin 1
NSD1	nuclear receptor binding SET domain protein 1
NUP98	nucleoporin 98kDa
OPN	osteopontin
PBX	pre-B-cell leukemia transcription factor
PKC	protein kinase C
PRC2	polycomb repressive complex 2
PRMT1	protein arginine N-methyltransferase 1
PTD	partial tandem duplication
pTEFb	positive transcription elongation factor b
RIME	immunoprecipitation-coupled mass spectrometry of endogenous protein
SAM	S-adenosyl-methionine
SELEX-seq	systematic evolution of ligands by exponential enrichment followed by sequencing
shRNA	short non-coding RNA
SMAD4	mothers against decapentaplegic homolog 4
TDG	thymine DNA glycosylase
TGFβ	transforming growth factor beta
WDR5	WD repeat protein 5
WT	wild-type
XPO-1	exportin-1
4C-seq	circularized chromosome conformation capture with high throughput sequencing
